# Epigenetic mechanisms of Immune remodeling in sepsis: targeting histone modification

**DOI:** 10.1038/s41419-023-05656-9

**Published:** 2023-02-11

**Authors:** Dan Wu, Yuxin Shi, Hao Zhang, Changhong Miao

**Affiliations:** 1 Department of Anesthesiology, Zhongshan Hospital, Fudan University, Shanghai, China; 2 Shanghai Key Laboratory of Perioperative Stress and Protection, Shanghai, China; 3 Department of Anesthesiology, Shanghai Medical College, Fudan University, Shanghai, China

**Keywords:** Post-translational modifications, Infection, Immunopathogenesis

## Abstract

Sepsis is a life-threatening disorder disease defined as infection-induced dysregulated immune responses and multiple organ dysfunction. The imbalance between hyperinflammation and immunosuppression is a crucial feature of sepsis immunity. Epigenetic modifications, including histone modifications, DNA methylation, chromatin remodeling, and non-coding RNA, play essential roles in regulating sepsis immunity through epi-information independent of the DNA sequence. In recent years, the mechanisms of histone modification in sepsis have received increasing attention, with ongoing discoveries of novel types of histone modifications. Due to the capacity for prolonged effects on immune cells, histone modifications can induce immune cell reprogramming and participate in the long-term immunosuppressed state of sepsis. Herein, we systematically review current mechanisms of histone modifications involved in the regulation of sepsis, summarize their role in sepsis from an immune perspective and provide potential therapeutic opportunities targeting histone modifications in sepsis treatment.

## Facts

- Histone modification is an essential part of epigenetic modifications, with generally revealed enzyme system and increasingly well-understood regulatory mechanism.
- Immune remodeling is one of the hallmarks of sepsis regulated synergistically or individually by various epigenetic factors.
- Histone modification pathway involves in the immune remodeling of sepsis and plays a role in different stages of septic immunity.
- Preclinical interventions targeting histone modifications have exhibited effectiveness in treating sepsis and relieving multiple organ dysfunction due to sepsis.

## Questions

- What are the individual sites of diverse histone modification in sepsis?
- What are the specific mechanisms of the crosstalk between different types of histone modifications or between histone modifications and other epigenetic modalities during immune remodeling in sepsis?
- Would therapies targeting histone modifications contribute to improved prognosis against immune imbalance in patients with sepsis?

## Introduction

Sepsis is a mortal response triggered by a severe infection [1]. In the clinic, sepsis is the leading threat to the lives of hospitalized patients and is the primary cause of death in the Intensive Care Unit [2]. As research on sepsis progresses, the understanding of sepsis has evolved from a concept of “systemic inflammatory response syndrome triggered by infection” (sepsis-1) to “severe and potentially fatal organic dysfunction caused by an inadequate or dysregulated host response to infection” (sepsis-3) [3]. This shift reflects the importance of a dysregulated host response in development of sepsis, in which the remodeling of immune cells plays an important role. In the early stages of sepsis, the body shows a pro-inflammatory host response, including the activation of inflammatory signals and a cytokine storm triggered by various cytokines [4]. As sepsis progresses to a later stage, the host response gradually becomes an immunosuppressive state. Sustained immunosuppression is associated with increased susceptibility to secondary infection and death in the clinic [5, 6].

Epigenetics is defined initially as a phenomenon that affects gene expression independently of base pairs alternation, which is reversible and heritable [7]. Subsequent studies have identified several cell-specific expression traits and environmentally mediated expression changes that affect gene transcription by modifying DNA, histones, and the extent of DNA packaging. Not all are heritable, but none alters gene sequence [8]. These discoveries have led to several significant research areas in epigenetic modification, including histone modification, DNA methylation, chromatin remodeling, and noncoding RNA [8]. Among them, histone modification is an inheritable epigenetic modification regulated by environmental factors (for example, metabolites [9]) and various histone modification transferases [10–12], as well as crosstalk with other epigenetic modalities. It plays a vital role in the development of disease.

Recent evidence suggests that epigenetic alterations receive widespread modulation during the progression of sepsis, with a crucial function in immunosuppression in the later stages of sepsis [13]. Different histone modification methods, including histone methylation, acetylation, O-GlcNAcylation, and lactylation, influence immune remodeling in sepsis through prolonged modulation of immune cells and are essential at all stages of sepsis development [6, 14]. As the exploration of histone modifications continues, the mechanisms associated with their involvement in sepsis continue to be elucidated.

In this review, we conduct an in-depth discussion on the immune remodeling process during sepsis from an epigenetic perspective emphasizing histone modification. We first briefly describe the immunity characteristics during sepsis, in the hyper-inflammatory phase of the early stages and the immunosuppressive state in the later stages. Then, we describe several traditional and newly discovered types of histone modifications and discuss new developments in their role in sepsis. In addition, we analyze the effects of different immune cells regulated by histone modifications in sepsis and the corresponding mechanisms and summarize the potential therapeutic options for targeting histone modifications in sepsis.

## An overview of the immune characteristics in sepsis

**Fig. 1:**
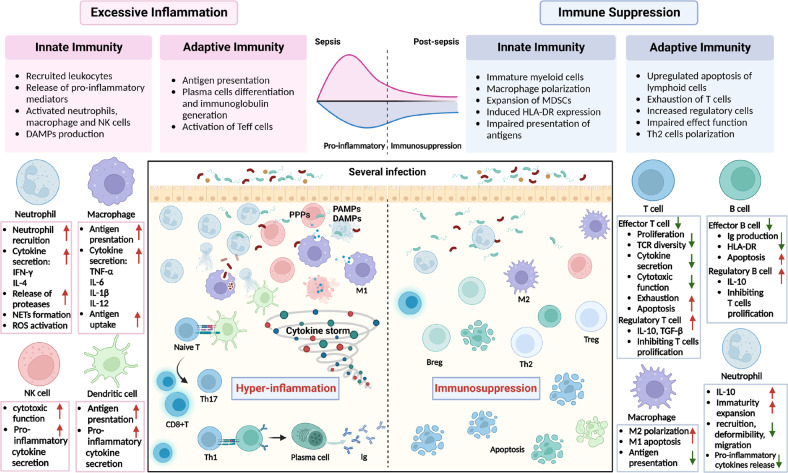
Hallmarks of the imbalanced immunity in sepsis. Sepsis manifests as an imbalance between inflammatory and anti-inflammatory responses, with a predominant inflammatory response in the early stages, referring to an excessive inflammatory state. In contrast, immunity exhibits long-term suppressive features in the late or post-septic stage. After the onset of severe infection, neutrophils are rapidly recruited. They release large amounts of pro-inflammatory cytokines, inducing a cytokine storm, and play a role in pathogen clearance. NK cells and macrophages enhance pathogen clearance and release cytokines to exacerbate inflammation further. Antigen-presenting cells enhance the ability to present antigens and promote the activation of effector T cells. Plasma cell differentiation is promoted, and the release of immunoglobulins is increased. During the immunosuppressive phase of sepsis, the number of innate and adaptive immune cells is reduced, and apoptosis is induced. Macrophages are polarized toward an anti-inflammatory phenotype. Simultaneously, circulating immature neutrophils are increased, and the inflammatory function of neutrophils is impaired. In adaptive immunity, the imbalance between effector and regulatory cells promotes immunosuppression, with the proportion and function of regulatory cells enhanced. DAMPs, damage-associated molecular patterns; PAMPs, pathogen-associated molecular patterns; PRRs, pattern recognition receptors; IL, interleukin; NETs, neutrophils extracellular traps; ROS, reactive oxygen species; T, T lymphocytes; B, B lymphocytes; NK, natural killer cells; Tregs, regulatory T lymphocytes; Th, T helper cell; Bregs, regulatory B lymphocytes; Ig, immunoglobulin; TGFβ, transforming growth factor-β; IFN, interferon; TNF, tumor necrosis factor; TCR, Toll-like receptor; HLA-DR, human leukocyte antigen DR.

Sepsis, defined as lethal organ dysfunction, is generally considered to be induced by severe infection and the dysregulated host response [6]. Once infection occurred, the innate immune system of the host stands in the breach to respond and senses the pathogens by recognizing pathogen-associated molecular patterns (PAMPs) and damage-associated molecular patterns (DAMPs) through diverse pattern recognition receptors (RRRs) to maintain host homeostasis via eliminating pathogens. While if overwhelming pathogens win the upper land, this response can be unbalanced and harm the host. Under this imbalanced condition, the immune response of patients with sepsis separates into two contrary states: excessive inflammation and immune suppression [15]. In the early stage of sepsis, pro-inflammatory and anti-inflammatory responses are activated, characterized by reprogramming genes in circulating leukocytes [14]. Other components, including vascular endothelial cells and platelets, act as regulators by cell death and releasing DAMPs, leading to organ damage and dysfunction [15]. Whereas with the progression of sepsis, the anti-inflammatory response is generally enhanced. As a result, clinical patients with sepsis always display immunosuppression, a state characterized by impaired antigen-presenting cells (APC) (including monocytes, dendritic cells, and B lymphocytes), increased anti-inflammatory mediators, and exhausted lymphocytes [16] (Fig. 1). In the clinic, profound and persistent immunosuppression induced by sepsis is tightly associated with secondary infections and increased mortality in patients [5, 17].

In the early stage of sepsis, Toll-like receptors (TLR), nucleotide oligomerization domain leucine-rich proteins, C-type, and mannan-binding lectin receptors, retinoic acid-inducible gene 1, and other common RRRs recognize DAMPs and PAMPs, then initiate immune responses [16, 18]. Translocation of nuclear factor-κB (NF-κB) is essential. It plays the core role in the induction of inflammation, which activates the target genes encoding pro-inflammatory cytokines, such as tumor necrosis factor (TNF), interleukin-1β (IL-1β), interleukin-8 (IL-8), triggering the “cytokine storm” [19]. Chemokines, including NO, intracellular adhesion molecule 1, vascular adhesion molecule 1, and ROS increase simultaneously [18]. Neutrophils act as the most abundantly recruited immune cells when infection occurs. Under the impact of chemokines, neutrophils migrate to the infection site to eliminate pathogens and assist injury healing by degranulation, phagocytosis, and release of cytokines [20, 21]. It also assists in clearing pathogens by forming neutrophil extracellular traps (NETs), a net-like structure consisting of DNA, histones, and serine proteases [22]. Neutrophils release pro-inflammatory cytokines, such as IL-1-β, IL-6, IL-12, and TNF-α, and release chemokines to recruit other immune cells. During the hyper-inflammatory period, NK cells act as important players, which regulate tissue cell necrosis and aggravate systemic inflammatory response by producing cytotoxic granules such as granzyme and perforin. It also releases cytokines and coordinates with myeloid via IFN-γ, especially macrophages [23]. Macrophages, as a dominant component of innate immunity, play roles in pathogens’ phagocytosis, antigen delivery, and inflammatory factors released in the surveillance against septic infection, promoting inflammation progress [24]. B cells act in the early stages of sepsis through antigen presentation and conversion to plasma cells for antibody production. Studies have also shown a novel role of B cells during the pro-inflammatory response in sepsis, with the identification of a subset--innate response activator B cells, which enhance the inflammation and production of monocytes and neutrophils by releasing interleukin-3 [25, 26].

The main characteristics of septic-immune suppression stages are lymphocyte exhaustion and apoptosis, and reprogramming of antigen-presenting cells [15]. CD4 + T cells, CD8 + T cells, NK cells, and follicular dendritic cells enhance apoptosis through death receptor- or mitochondrial-mediated pathways [6, 27, 28]. An imbalanced condition of effect function and regulatory function exists in lymphocytes, with a proportion of regulatory and exhausted B cells and T cells elevated [17, 29]. These regulatory cells release anti-inflammatory cytokines, such as IL-10 and TGF-β, and inhibit T cell proliferation. Evidence demonstrates the increased programmed cell death receptor-1 (PD-1) and its ligand (PD-L1) on T cells under septic conditions [30]. Besides, patients with sepsis showed upregulation of CD4^+^ T helper 2 cells and reduced TH1 and TH17 cells [31]. B cells are also involved in developing an immunosuppressive status, and downregulated human leukocyte antigen (HLA)-DR reduces their antigen-presenting ability. At the same time, the differentiation of B cells to plasma cells is blocked due to reduced TH1 cells, resulting in decreased immunoglobulin secretion [32]. In a cecal ligation and puncture (CLP) model, a subset of B cells (CD39^HIG^) inhibits macrophage function by adenosine and promotes immune suppression, indicating that B cells can regulate other immune cells in sepsis through a metabolic way [33]. Macrophage reprogramming is another feature of immune suppression. With the progression of sepsis, the polarization of macrophages to an M2-like phenotype leads the inflammatory state to an immunosuppressive state, characterized by reduced pro-inflammatory cytokines secretion, upregulated anti-inflammatory mediators, and impaired pathogens phagocytosis capacity [24]. Declined HLA-DR expression on monocytes and macrophages is a hallmark of immunosuppression in sepsis [34]. Studies have also shown that sepsis-induced systemic inflammation impairs DCs proliferation and reduces the expression of HLA-DR, with antigen-representing capacity decreasing [35]. In addition, immature neutrophils with low expression of CD10 and CD16 have been found in post-sepsis patients, and these cells exert their immunosuppressive functions by suppressing the proliferation of T lymphocytes [17]. In general, immune cells act dynamically and play diverse roles in different stages of sepsis, regulated by signals including cytokines, intercellular interactions, and metabolic remodeling.

Recent studies have verified that epigenetic regulation is critical during the septic-immune process, which participants both in the excessive inflammation and later-stage immunosuppressive response [6, 14], and indicate histone modifications act as one of the dominant players [15]. Next, we focus on the various histone modifications and introduce the concrete mechanisms function in sepsis.

## Histone modifications in sepsis

**Fig. 2:**
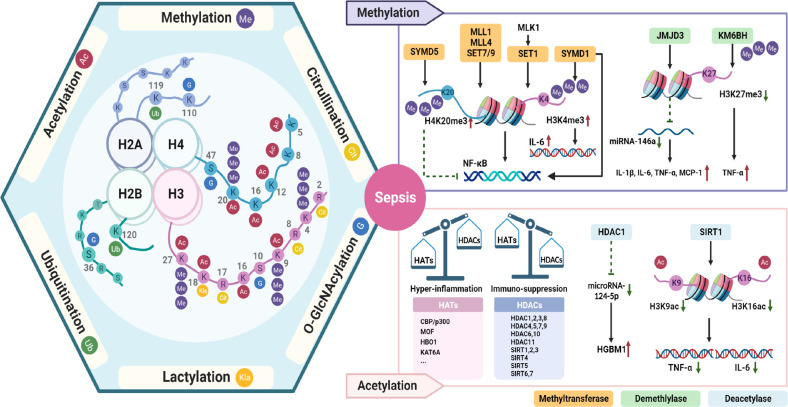
Histone modifications in sepsis. Four core histones, H2A, H2B, H3, and H4, undergo histone modifications at different amino acid sites in response to specific enzymes, including histone methylation, acetylation, citrullination, O-GlcNAcylation, ubiquitination, and lactylation. Differential histone modification modalities and their regulatory enzymes have been shown to play a role in sepsis or endotoxemia caused by LPS. As two of the more thoroughly studied modalities of histone modifications, the regulatory mechanisms of histone methylation, acetylation, and their catalyzing enzymes in sepsis are better explained. MLL, mixed-lineage leukemia 1; MKL1, megakaryocytic leukemia 1; MCP1, monocyte chemotactic protein-1; JMJD3, Jumonji domain-containing protein D3; KM6BH, (K)-specific demethylase 6B; SIRT, sirtuin; HAT, histone acetyltransferases; HADC, histone deacetylases; NF-Κb, nuclear factor-κB; HGMB1, high mobility group box 1; TNF, tumor necrosis factor; IL, interleukin.

The basic components of eukaryotic chromatin include histones and nucleosomes composed of histones and DNA. Diverse post-translational modification processes are often present in the tail regions of these histone proteins [36]. Histone modifications play a role in various biological processes by regulating the transcriptional activity of genes through different mechanisms. The types of histone modifications include acetylation, methylation, ubiquitination, etc., with methylation and acetylation being the most common [11]. Novel histone modifications have been proposed in recent years, such as histone glycosylation and histone lactylation. Here we describe histone methylation, acetylation, ubiquitination, O-GlcNAcylation, lactylation, and citrullination and discuss new findings on the relationship between different modifications and sepsis (Fig. 2).

### Methylation and demethylation

Histone methylation is an important form of post-transcriptional modification widespread in organisms. It plays a key role in health and disease states, occurring on all resides, including arginine, lysine, and histidine [12]. Protein methylation can occur at the side chain or amino terminus of amino acids. In histones, lysine and arginine residues are the most common sites for methylation modifications. They have diverse effects on gene activity depending on the specific residues modified, the extent and pattern of methylation, and the genomic context in which methylation occurs. Of all histones, histone H3 is the major site of histone methylation, although other core histones can also be methylated [37]. During this process, S-adenosine methionine acts as a substrate and transfers its methyl to lysine residues of the histone under the catalysis of histone methyltransferase (HMT, also called “writers”) [11]. The removal of methyl is regulated by histone demethylases (HDMs; also called “erasers”) [10]. On the ε-amine group of lysine, it can be mono-, di-, or trimethylated, referred to as me1, me2, and me3 [10]. Some of the more widely studied methylated histones include histone H3 lysine 4 (H3K4), H3K9, H3K27, H3K36, H3K79, and H4K20 [12]. In general, H3K4, H3K36, and H3K79 play activating roles and are principally present in transcriptionally active gene regions in chromatin. On the contrary, H3K9, H3K27, and H4K20 are mainly associated with gene expression silencing and often act as repressive markers [6, 11].

The relationship between histone methylation and sepsis continues to be explored as research into histone modifications is conducted. Several studies demonstrate that histone methylation may have a long-lasting effect on sepsis [6, 38]. In sepsis, multiple methyltransferases induce changes in inflammatory signaling and related inflammatory factor expression by catalyzing histone methylation. NF-κB is widely recognized as a master playing a regulatory function in the pro-inflammatory transcription process. Mounting evidence has identified the importance of histone methylation in NF-κB-dependent inflammatory responses [39]. Histone H3K4 methyltransferases, including mixed-lineage leukemia 1(MLL1), MLL4/WBP7, and SET7/9, are required for transactivation in NF-κB targeting genes [39]. Megakaryocytic leukemia 1 defends the landscape of H3K4me3-mediated inflammatory response activated by NF-κB signaling, with its role of recruiting SET1, an H3K4 trimethyl transferase, to the promoter regions of p65 target genes [39]. Long-term inflammatory damage and impaired wound healing due to sepsis are orchestrated by downregulation of the methyltransferases MLL1 and H3K4me3 on inflammatory gene sites of NF-κB in the bone marrow [38]. In vitro, lipopolysaccharide (LPS) mediates the upregulation of lysine methyltransferase SYMD1 expression in endothelial cells, which increases IL-6 release by promoting H3K4me3 of IL-6 promotor. This process is independent of the mechanism by which SYMD1 promotes NF-KB-mediated inflammatory activation [40]. In contrast, SMYD5-mediated methylation of H4K20 is demonstrated as a repressive feature that prevents transcription of downstream genes of TLR4 signaling for pro-inflammation [41]. Jumonji domain-containing protein D3 (JMJD3), a kind of H3K27 demethylases, acts as a pro-inflammatory player in sepsis [42, 43]. In the early stages of sepsis in mice, JMJD3 negatively regulates the transcription of miR-146a, an anti-inflammatory micro-RNA, through demethylation of the miR-146a promoter. It enhances the production of pro-inflammatory cytokines such as IL-1β, IL-6, TNF-α, and monocyte chemotactic protein-1, which promotes septic mice’s early-stage death. Meanwhile, miR-146a transcription is positively regulated by NF-κB p65, which promotes anti-inflammatory responses by inhibiting the binding of JMJD3 to the miR-146a promoter [43]. Endothelial progenitor cell-derived extracellular vesicles containing miR-93-5p exert a protective effect on endothelial cells by regulating the (K)-specific demethylase 6B (KDM6B lysine)/H3K27me3/TNF-α axis in sepsis-induced acute kidney injury [44]. The above results suggest a regulatory relationship between the histone methylation process and noncoding RNAs. Methylated histones can act as both upstream or downstream signals of noncoding RNAs to mediate the regulation of inflammatory signals associated with the sepsis process.

### Acetylation and deacetylation

Acetylation is a reversible post-translational modification of proteins, catalyzed by histone acetyltransferases (HATs), histone deacetylases (HDACs) for the transfer of the acetyl portion to lysine residues, and the removal of the acetyl group, respectively. HDACs include Zn^2+^-dependent HDACs and NAD^+^-dependent sirtuins (SIRTs) [45]. Histone 1 is the most common protein to undergo acetylation. The presence of acetylation modifications in the four typical core histones H2A, H2B, H3, and H4 is also widely recognized [45]. Histone 3 and histone 4 acetylation, including H3K9ac, H3K14ac, H3K18ac, H3K23ac, H3K27ac, H3K56ac, H4K5ac, H4K8ac, H4K16ac, and H4K20ac, are catalyzed by HATs such as CBP/p300, MOF, HBO1, or KAT6A [46]. 18 types of deacetylases were discovered in humans, including HDAC1-11 and SIRT1-7 [11]. Acetylated lysine of histones usually supports transcription. Numerous studies have revealed the correlation between histone acetylation levels and the expression of pro-inflammatory cytokines and other anti-microbial products, with acetylases and deacetylases playing an important role [47]. Exploring the process of histone acetylation and the enzymes connected is essential for understanding the mechanism of sepsis progression and the immunosuppressive state.

The balance between HATs and HDACs is maintained in healthy individuals, but sepsis can disrupt this homeostasis [46]. When sepsis begins, upregulation and activation of HATs open chromatin structure and promote transcription of a large number of pro-inflammatory genes, including TNFα, IL-1β, and iNOs [48]. Likewise, HDACs also inhibit the expression of anti-inflammatory factors, further promoting inflammation [49, 50]. As sepsis progresses, gene silencing and chromatin structural remodeling caused by HADCs trigger the alternation of cellular expression files, which are assumed to be associated with immunosuppression in late sepsis [46, 51]. Classical histone deacetylases, including HDAC1-8, act as players in the progression of sepsis and inflammation and are involved in the multi-organ dysfunction of sepsis [46, 49, 52, 53]. These deacetylases regulate the expression of inflammatory genes on immune cells or cellular apoptosis in polymicrobial CLP septic models or LPS-mediated endotoxemia models [46, 53]. The role played by the deacetylase SIRT1 in sepsis also received wide recognition [54]. During endotoxin tolerance in sepsis, histone deacetylase SIRT1 accumulates on the IL-1β and TNF-α promoters, and NAD^+^ levels are elevated, resulting in enhanced H3K16 deacetylation [54]. It was demonstrated that SIRT1 reduces H3K16 acetylation and inhibits TNF-α transcription during sepsis-induced inflammation by targeting the TNF-α promoter [55]. Zhang et al. found that SIRT1 reduced the acetylation of histone H3K9 in the IL-6 and TNF-α promoters and blocked their expression [56]. Studies also illustrated the mechanisms by which histone acetylation affects sepsis through indirect regulation of gene expression by acting on microRNAs. Nong et al. revealed that HDAC1 exacerbated myocardial damage in septic mice by causing an increase in HMGB1 through the downregulation of microRNA-124-5p [49]. In a TC-1 model with Trichostatin A (TSA) or 5-Azacytidine intervention, IL-6 increased histone acetylation by promoting the glycolytic process causing the accumulation of Acetyl-CoA accumulation. The increased acetylated histones promoted the progression of sepsis by upregulating microRNA-29a to inhibit STAT3 [57].

### Citrullination

Citrullination refers to the process by which peptidylarginine residues are converted to pepetidycitrulline residues through deimination with the catalysis of calcium-dependent enzyme peptidyl arginine deiminases (PADs), acting as a crucial avenue of protein modification, including histones [58]. To date, four types of PADs have been discovered in humans. Emerging evidence has shown the importance of citrullinated histone H3 (CitH3), mainly catalyzed by PAD4, for promoting a rapid form of NET formation (NETosis) and NETosis-mediated diseases, including sepsis [59]. Accumulating studies have revealed the link between CitH3 and sepsis. In LPS-induced septic models of mice, circulating CitH3 levels are verified to be a diagnostic marker for early sepsis and can reflect the severity of sepsis [60]. This has also been demonstrated in patients with sepsis since a correlation between high serum CitH3 and poor prognosis of sepsis is elucidated [61–63]. Novel research into systemic dysfunction in sepsis also identified a higher circulating CitH3 level in serum [64–66]. Tian et al. implicated that elevated CitH3 is correlated with sepsis-induced acute respiratory distress syndrome and lung dysfunction in patients. In mechanism, CitH3 activates caspase-1-dependent inflammasome in bone marrow-derived macrophages and bone marrow-derived dendritic cells and induces acute lung injury (ALI) [65].

### Ubiquitination

Histone ubiquitination, including mono- and poly-ubiquitination, is considered an essential player for genome stability, orchestrating various processes on chromatin. Among core histones, mono-ubiquitination prevalently exists and plays a core role in cellular DNA damage [67]. A sequential enzymatic reaction induces the occurrence of mono-ubiquitination: ubiquitin is first activated by a ubiquitin-activating enzyme(E1) in an ATP-dependent manner, then binds to cysteine residues on a ubiquitin-conjugating enzyme (E2) by a thioester bond, and finally, ubiquitin in E2 is transferred to a target lysine site in the histone by RING finger ubiquitin ligase E3 [68]. Histone H2A lysine 119 mono-ubiquitination (H2AK119ub) and H2BK120ub are the two major histone mono-ubiquitination events [68]. Although rare, poly-ubiquitinated histones also occupy a position during the DNA repairing process [69]. Like other histone modifications, ubiquitination of histones is reversible and tightly regulated by histone ubiquitinating and deubiquitinating enzymes. Currently, studies on histone ubiquitination in sepsis are limited and inflammatory immunity. Ubiquitin-specific peptidase 22 is upregulated in sepsis-induced myocardial dysfunction [70]. A recent study has elucidated the role of the deubiquitinating enzyme USP38 in innate immunity to inflammation [71]. In a mouse model of endotoxemia, investigators identified USP38 as a novel histone deubiquitinase that removes monoubiquitin on H2B at lysine 120 specifically and recruits the demethylase KDM5B to the promoters of the proinflammatory cytokines IL6 and IL23a. KDM5B inhibits the binding of NF-κB transcription factors to the IL-6 and IL-23a promoters by reducing H3K4 trimethylation [71].

### Lacylation

In the clinic, circulating lactate level in serum is often considered an essential indicator of the severity of sepsis and shock. Recent studies on metabolite intermediates have revealed the importance of lactate and its regulatory functions in inflammatory and immune responses [72]. The codes generated by posttranslational histone modifications act as critical sensors of metabolism and link metabolic changes to stable gene expression [73]. With an in-depth eye on lactate and histone, Zhang and his colleagues identified a novel type of epigenetic modification, lactate-derived histone lysine lactylation, by mass spectrometry and ^13^C glucose tracer. They proposed that this histone modification, existing on 28 sites of core histones, directly stimulates gene transcription in chromatin, holding different temporal dynamics from acetylation, and impacts gene expression of immune homeostasis under infection. As a common acetyltransferase, P300 plays the role of L-la introduction during lactylation [9]. In another study, they further investigated the regulatory enzymes of this process and reported class I histone deacetylases (HDAC1–3) as histone lysine delactylases [74]. A clinical trial done by Chu et al. has reflected the association between H3K18 lactation and poor prognosis in patients with sepsis. H3K18la may enhance the overexpression of inflammatory cytokines, including IL-2, IL-5, IL-6, IL-8, IL-10, IL-17, IFN-α, and Arg in patients, and promote the occurrence of macrophage anti-inflammatory response in sepsis [75]. Clinical and preclinical studies have shown the potential value of histone lactylation in sepsis. High mobility group box 1 protein (HMGB1), a nuclear protein, plays a vital role in the late immune response to sepsis [4]. It has been reported that lactylated HMGB1 increases and is secreted by macrophages via exosomes and promotes endothelial cell permeability under septic conditions, which declared that lactylation also impacts sepsis in a non-histone way [76]. Intriguingly, new findings reveal that lactylation is associated with a variety of epigenetic modifications, including acetylation [9, 76] and M^6^A methylation [77, 78]. In cancer, histone methylation upregulates the expression of METTL3 [77], which mediates m6A, and YTHDF2 [78], which recognizes m6A, thereby promoting cancer progression. Although these pathways have not been demonstrated in sepsis, these results may provide new ideas for the mechanism of histone lactylation regulation in sepsis progress.

### O-GlcNAcylation

O-GlcNAcylation is a type of histone modification that adds o-GlcNAc to Ser and Thr through O-linage, controlled by a pair of enzymes: o-GlCNAc transferase (OGT) and O-GlcNAc enzyme. These enzymes catalyze the transfer of GlcNAc to the hydroxyl groups of target amino acid residues and the hydrolysis of sugar modification, respectively [79]. Accumulating studies have pointed O-GlcNAcylation plays a crucial role in chromatin remodeling and regulation of gene expression as part of the “histone code”. O-GlcNAcylation occurs on histone H2A Thr101, histone H2B Ser36, H4 Ser10, and histone H4 Ser47. Notably, O-GlcNAcylated histones regulating the levels of other histone modification, with increased H3K4me3 and reduced H3K9me3 are verified [80]. Its biological functions are exerted directly through histone modification or indirectly by acting on other histone modification enzymes [80]. During diverse pathological conditions, O-GlcNAc signaling is sensitive to various forms of stress-containing sepsis and shock [79]. Glucosamine is verified to reduce inflammation infiltration and lung injury after sepsis or LPS stimuli through modulation of O-GlcNAcylation of nucleocytoplasmic proteins in two disparate models [81, 82]. In early-stage sepsis, O-GlcNAc acts as a cardiovascular protector by restoring SERCA2a expression without impacting inflammatory responses [83]. Similarly, O-GlcNAc stimulation was also found to improve the prognosis in young rats with sepsis, and O-GlcNAcylated proteins are mainly correlated with cellular metabolism [84]. Whereas, this beneficial impact of O-GlcNAc is independent of changes in gene profile expression at the early stages of sepsis [85]. A recent study has illustrated an O-GlcNAcylation-mediated mechanism of innate immune regulation in sepsis progress. LPS inhibits the binding of RIPK3-RIPK1 and RIPK3-RIPK3 by decreasing OGT-mediated RIPK3 T467 O-GlcNAcylation and suppresses the downstream innate immune response and necroptotic signaling [86]. This study first shed light on the relationship between O-GlcNAc signaling and immunity in sepsis, suggesting that O-GluNAcylation might involve immune-related mechanisms during the development of sepsis.

## Histone modifications in sepsis immunity and progression

**Fig. 3:**
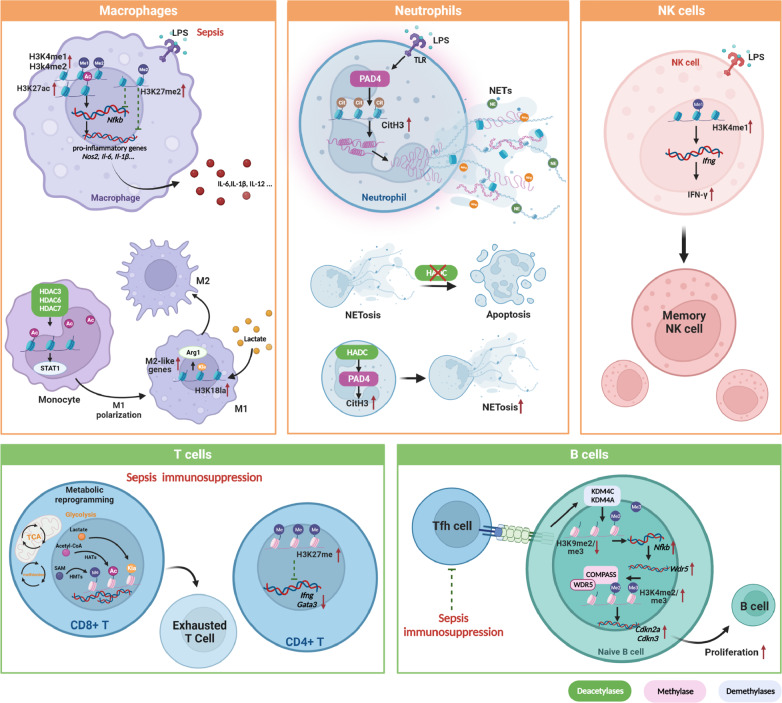
Histone modifications regulate immunity remodeling in sepsis. In sepsis or endotoxemia models constructed by LPS, histone modifications are involved in the functional regulation of innate immunity and adaptive immunity. Specifically, histone modifications regulate polarization and inflammatory factor secretion in macrophage and NETs production and shift of death paradigm in neutrophils, as well as the generation of memory NK cells. In adaptive immunity, histone modifications act as players in forming exhausted T cells. Simultaneously, it is associated with the process of Tfh-induced B cell proliferation. LPS, lipopolysaccharide; IL, interleukin; NETs, neutrophils extracellular traps; T, T lymphocytes; B, B lymphocytes; NK, natural killer cells; Tfh, T follicular helper cell; IFN, interferon; TCR, Toll-like receptor; TCA, tricarboxylic acid.

The immunity is modulated and reshaped during the progression of sepsis. Substantial evidence suggests that histone modifications are involved in this remodeling process, both adaptive and innate immunity, influencing cell differentiation, mode of death, inflammatory function, etc (Fig. 3).

### Innate immunity

#### Macrophages/monocytes

During the hyperinflammatory phase of sepsis, monocytes/macrophages act to monitor the infection by phagocytosing bacteria, presenting antigens, and producing large amounts of cytokines [6]. Macrophage and monocyte reprogramming is a key feature of immunosuppression in sepsis, exhibiting a decreased ability to release pro-inflammatory cytokines after stimulation of LPS, TLR agonists, or various bacterial compounds [17]. Diminished monocyte HLA-DR expression is another characteristic of the immunosuppressive phase of sepsis and is associated with poor clinical patient prognosis [87, 88]. Macrophage polarization is a double-edged sword in sepsis. M1-like macrophages are usually considered classically activated, exhibiting pro-inflammatory properties during host infection, while the other terminal of macrophage activation, M2-like macrophages, mainly exert anti-inflammatory functions [89]. The over-activation of M1 cells in the early stage of sepsis makes targeting the shift of M1-like macrophages to the M2 phenotype a potential therapy. In contrast, M2 polarization and the release of large amounts of IL-10, TGF-β, and various anti-inflammatory mediators during the later phase of sepsis induce immunosuppression and the development of secondary infections [24]. Mounting evidence suggests the involvement of histone modifications in the activation of M1 and M2 macrophages, respectively, and macrophage polarization [90].

Histone modifications differentially regulated genes of macrophages during sepsis and change over time, which impacts macrophage behaviors. Epigenomic analysis of macrophages after LPS stimulation presents a molecular landscape in LPS-induced immune tolerance through a time-dependent approach. H3K27ac with subsequent H3K4me1 occurs in diverse levels in genes involved in cytokine response and NF-kB signaling [91]. In murine monocytes challenged by different doses of LPS, genome-wide H3K27ac modification, and gene expression were analyzed by epigenomic and transcriptomic sequencing. The differences between H3K27ac on gene enhancers between low inflammation and severe exhaustion point to the importance of epigenetic regulation in driving different inflammatory responses [92]. In early sepsis, the H3K27me3 demethylase JMJD3 promotes inflammation by negatively regulating the transcription of anti-inflammatory microRNA-146a (miR-146a) in peritoneal macrophages of septic mice [43]. H3K4me3 acts an important role during LPS-induced endotoxemia and influences macrophage plasticity [93]. The epigenetic profiles of H3K4me3 level under different stimuli (IFNγ + LPS or IFNγ + LPS + immune complex (IC)) show global up-regulated H3K4me3 enrichment in IFNγ + LPS + IC group [93]. A novel study showed that non-methylation transferases also play a role in macrophages’ histone methylation-mediated pro-inflammatory gene regulation [93]. RUVBL1/2, a member of the AAA + (ATPase associated with diverse cellular activities) superfamily of ATPases, promotes macrophage pro-inflammatory response by upregulating histone H3K4me3 levels and recruiting NF-κB to the enhancer of Nos2 and IL-6 promoter [94]. Post-sepsis bone marrow-derived macrophages and wound macrophages (CD11b+, CD3-, CD19-, Ly6G-) exhibit decreased expression of inflammatory cytokines essential for wound healing (IL-1β, IL-12, and IL-23), which is mediated by reduced expression of MLL1 and decreased H3K4me3 at NFκB binding site on genes related to inflammation [38]. Additionally, levels of H3K9me2 increase in genes’ promoter region encoding the pro-inflammatory factors IL-1β and TNF in macrophages after LPS-induced tolerance, reducing the inflammatory response [15]. HDAC3 is critical to the inflammatory gene expression program of macrophages stimulated by LPS [95]. Correspondingly, upregulation of HDAC2 expression was found in macrophages after LPS stimulation. Knockdown of HDAC2 inhibits the transcription of pro-inflammatory genes, including IL-12, TNF-α, and iNOS. In mechanism, HDAC2 promotes macrophage inflammation by inhibiting Jun-c and indirectly enhancing inflammatory gene transcription [50]. HDACs are also involved in macrophage polarization as key regulators. HDCA3 and HDAC6, as well as HDAC7, regulate the polarization of macrophages into an M1-like phenotype [24, 90]. These depend on abundant inflammatory gene activation, with STAT1 acting as the primary signal [96]. Newly identified histone lactylation in macrophages upregulates genetic loci associated with M1 macrophage polarization in the late phase, including Arg1, which regulates macrophage behavior and promotes alternation of macrophages to reparative M2 macrophages [9].

Additionally, metabolites are involved in the regulation of macrophages during sepsis via the histone methylation pathway. The glutamine metabolite, α-ketoglutarate, regulates M2-like macrophage gene promoter via Jmjd3-dependent H3K27 demethylation [97]. This improves oxidative phosphorylation and fatty acid oxidation and promotes the activation of M2-like macrophages [98]. Yu et al. elucidated that one-carbon and glucose metabolism promotes pro-inflammatory macrophage in LPS-induced inflammation through modulating S-adenosylmethionine (SAM) and reprogramming histone methylation. H3k36me3 acts as a possible epigenetic target, which is sensitive to LPS stimulation and controls SAM availability [99]. γ-amino butyric acid (GABA), a kind of neurotransmitter, orchestrates macrophage maturation and pro-inflammatory responses. In mechanism, GABA-induced H3K4/9me2 demethylation regulates LSD1-BCL2L11/DUSP2 signaling and further limits IL-1β production [100].

#### Neutrophils

Neutrophils, as mentioned earlier, are an important component of innate immunity, which promotes hyperinflammation early in sepsis by releasing proteases, reactive oxygen species (ROS), and NETs. While during the immunosuppressive phase, neutrophils exhibit various immune impairment features, including decreased migration, reduced intracellular myeloperoxidase and lactoferrin levels, and diminished oxidative burst capacity [6]. Novel studies have shown the link between histone modification and regulation of neutrophils. The role of HDAC11, a recently discovered member of the HDAC family, in the differentiation and maturation of neutrophils has been elucidated. As neutrophils differentiate and mature, HDAC11 is produced in large quantities. Meanwhile, the level of HDAC11 correlates with the function of neutrophils. Neutrophils lacking HDAC11 are better able to cope with LPS-induced sepsis, granulocyte expansion, and increased extramedullary hematopoiesis, showing increased migration of inflammatory cytokines and phagocytosis [101, 102]. The use of different concentrations of HDAC inhibitors (HDACis), belinostat, and panobinostat, to promote histone acetylation revealed a shift in the type of neutrophil death from NETosis to apoptosis. Hamam et al. illustrated that histone acetylation level could change the neutrophil death mode [103]. The above results suggest that histone acetylation may play a role in all physiological stages of neutrophils and can affect their function. Increased H4K16ac level is functionally associated with neutrophil apoptosis [104]. In addition, epigenetic regulations involve in the immuno-training process of neutrophils. In a neonatal mouse model, induction of trained immunity using Bacillus Calmette-Guerin (BCG) plus bacterial lipoprotein (BLP) promotes inflammatory mediators release, increases neutrophil recruitment, and accumulates bacterial clearance, thereby protecting mice against cecal slurry peritonitis-induced polymicrobial sepsis. Mechanically, BCG + BLP stimulation leads to enhanced H3K4me3, H3K27Ac, and reduced H3K9me3 at the promoter of inflammatory and antimicrobial genes [105].

##### NETs and NETosis

During sepsis, circulating neutrophils migrate to the site of infection to assist in pathogen clearance by releasing NETs, which consist of extracellular chromatin of CitH3, myeloperoxidase, and other intracellular molecules excreted by neutrophils [106]. This is a specific form of neutrophil death in sepsis. Although NETs contribute to clearing pathogens, excessive NET formation can promote inflammation and cause tissue damage [107]. Here, we introduce the effects of histone modifications on NET in sepsis.

Histone citrullination induced by PAD4 in neutrophils and subsequent chromatin densification are hallmarks of NETs induction. Direct evidence on the role of other histone modifications in neutrophils and NETosis is limited [59]. Stable levels of H3K9, H3K27, and H3K36 methylation are displayed in human neutrophils, but when NETotic agonists are utilized, mouse cell line-derived neutrophils no more show this characteristic. Based on these studies, the investigators hypothesized that induction or inhibition of histone methylation might alter NETs formation [59]. Recently, researchers have come to understand the role played by histone acetylation in NETs. Acetylation of histone promotes the formation of NETs [108]. A recent study by Poli et al. showed that HDACs play a key role in inducing NETosis in human and mouse neutrophils. HDACs act as a zinc-dependent lysine deacetylase by catalyzing the deacetylation of histone H3, thereby enabling the activity of PAD4 and the formation of NETosis. Notably, inhibition of HDAC prevented microbial pneumonia and infectious shock in mice and reduced NETosis and inflammation. This finding elucidates the potential role of histone deacetylation in sepsis and NETosis [109]. An interaction exists between histone acetylation and citrullination. PAD4 was shown to be dependent on HDAC1 activity. Knockdown of HDAC1 resulted in reduced levels of PAD4 and CitH3 [110, 111]. These suggest that other histone modifications may affect neutrophils and NETs through an indirect mechanism.

#### NK cells

In sepsis, NK cells exert an immune memory phenomenon similar to adaptive immune cells, responding to various infections and immunogens regulated by immunological education or licensing [112]. Novel research suggests an important role of histone methylation in the generation of long-term immune memory in NK cells [113]. The researchers found that LPS-induced systemic inflammation edited NK cells in mice to produce a memory signature that persisted long after endotoxemia. Transfer of memory NK cells to naive mice enhanced their anti-infective immunity. In mechanism, upregulation of H3K4me1 at the -22kb site of ifng promotes IFN-γ expression and mediates the generation of memory-like NK cells [113].

### Adaptive immunity

#### T cells

T cell exhaustion is considered a hallmark of suppression in sepsis. During T-cells exhaustion, alternations of metabolism crosstalk with histone modifications show that imbalanced intermediates caused by poor metabolic fitness participate in different processes, including histone acetylation and methylation [114]. Both mitochondrial metabolism and glucose intake are suppressed in exhausted T cells, leading to the downregulation of metabolites such as acetyl-CoA and SAM. In turn, these metabolites play an essential role in the histone modification process, modulating different gene expressions of T cells by affecting different enzymes, for instance, SIRT1, HAT, and JMJD3 [114]. In the severely septic mice model, histone methylation is regulated in the promoter region of the gene encoding GATA3 and T-box protein 21 (TBX21; also known as TBET), thereby promoting CD4^+^T cell inactivation and dysfunction [115]. Lai et al. have recently reported the role of protein arginine N-methyltransferase4 (PRMT4) (also known as coactivator-associated arginine methyltransferase 1), a chromatin modulator, during the septic immunosuppressive process, which reveals a pathway of epigenetic enzyme induced lymphocytes deaths. Upregulated expression of PRMT4 is found in both lymphocytes and monocytes under LPS stimuli or sepsis, whereas it shows more pronounced in activated T cells and explicitly facilitates lymphocytes apoptosis via caspase 3 signaling [116].

#### B cells

During sepsis, B cells first display pro-inflammatory function right after infection [17]. As sepsis progresses, the main functions of B cells, presenting antigens to T cells and producing the antibody, are impaired. Although the exact alternations of B cells in sepsis have not been fully decoded, compromised antigen-specific antibody production and increased apoptosis are considered characteristics of septic immune suppression [32]. Evidence shows that histone modifications are associated with B cell activation and differentiation [117]. After LPS stimuli, levels of H3K9me2/me3 and H3K4me2 are all regulated in B cells [117]. Tsai et al. revealed that WDR5, an MLL complex member facilitating H3K4 methylation, links these two histone modifications and stimulates the proliferation of B cells. They conducted their study in a B cell model with T follicular helper (Tfh) cells mimicking -signaling activation. H3K9me3/me2 is removed via KDM4A and KDM4C and cooperates with NF-κB to upregulate Wdr5 expression. Meanwhile, WDR5 acts as a core subunit of the COMPASS histone H3K4 methyltransferase complex, increasing H3K4me3/me2, thus promoting the transcription of Cdkn2a and Cdkn3, then regulates the proliferation of stimulated B cells [118]. Clinical data on sepsis suggest that immunosuppressed patients exhibit reduced circulating Tfh numbers and impaired B-cell maturation [119]. Simultaneously, mouse models have shown polymicrobial sepsis to impede Tfh-mediated maturation of B cells [120]. Histone modifications may explain the impaired function of Tfh in B cell maturation. In a CLP mouse model, the HDAC6 inhibitor, Tubastatin A, was shown to restore B lymphocyte percentage, as well as other innate immune cells, to improve sepsis prognosis [121], which suggests the potential role of histone deacetylation to B cell down-regulation in sepsis.

## Treatments targeting histone modifications in sepsis

To date, inhibitors targeting HDACs in sepsis have been extensively studied [46, 103], while interventions for other histone modifications are still limited (Table 1). Li et al. first illustrated suberoylanilide hydroxamic acid (SAHA) as a histone deacetylase inhibitor to protect mice against LPS-induced septic shock. SAHA reversed the downregulation of histone acetylation caused by LPS, reduced TNF-α and IL-6 expression, and improved long-term survival after LPS stimuli [122, 123]. Their further study reveals SAHA also inhibits CitH3 triggering, which is associated with a better septic prognosis [124]. As a broad-spectrum Inhibitor, SAHA protective function targets all classes of HADCs. Its role in sepsis to prevent distant organ damage and alleviate coagulation imbalances has also been discovered in CLP models [125, 126]. Similarly, HDACi TSA has a protective effect during sepsis [127–129]. In macrophages, TSA modulates macrophage polarization toward the M2 phenotype through enhanced autophagy to reduce systemic inflammation and prevent mice with polymicrobial sepsis from death [130]. In addition, TSA impedes the induction of endotoxin tolerance [131]. Other pan-HDACs contain valproic acid, KBH-A42, and butyric acid, which reduce pro-inflammatory gene expression and organ damage in sepsis [46, 132–135]. However, the clinical application may be limited by their proven toxic effects [46, 136]. The HDAC6--specific inhibitor tubastatin A mediates the regulation of immune cells in sepsis [115, 137]. The elective Sirt1 inhibitor EX527 [138] and the selective Sirt2 inhibitor AGK2 [139] improve the outcome of lethal septic mice through reduced IL-6, TNF-α, and coagulopathy as well. Intriguingly, there is evidence that EX527 also exerts a role in reversing the hypoinflammatory state in the late stages of sepsis and effectively reduces mortality in CLP mice [54, 140]. EX527 intervention reverses immune tolerance in effector lymphocytes and in innate immunity and leads to a diminished ratio of CD4 + Treg to activated CD4 + T cells [54, 141].

**Table 1:** Potential treatments targeting histone modifications in sepsis.

Potential Target		Inhibition	Effects	Timing	ref
Histone Deacetylation	HDACs	SAHA	long-term survival ↑ , coagulation imbalance ↓ , organ injury ↓ , TNF-α ↓ , IL-6 ↓ , CitH3 triggering↓	H	[122–126]
		TSA	survival ↑ , TNF-α ↓ , MPO ↓ , IL-1β ↓ , IL-6 ↓ , IL-10 ↓ , ICAM-1 ↓ , neutrophil infiltration ↓ , M2-like polarization↑	H	[127–130]
			endotoxin tolerance ↓ , IL-6 ↑ , IL-10↓	I	[131]
		VPA	MPO ↓ , TNF-α ↓ , IL-1β ↓ , NO ↓ , iNOS↓	H	[134, 135]
		Butyric acid	MPO ↓ , TNF-α ↓ , IL-1β ↓ , NO ↓ , iNOS↓	H	[132]
		KBH-A42	survival ↑ , organ injury ↓ , IL-6 ↓ , ICAM-1 ↓ , neutrophil infiltration↓	H	[133]
	HDAC6	TubA	survival ↑ , organ injury ↓ , MPO ↓ , TNF-α ↓ , IL-6 ↓ , B cells ↑ , innate immune cells ↑ , bacterial clearance↑	H	[131, 137]
	SIRT1	EX527	survival ↑ , coagulation imbalance ↓ , TNF-α ↓ , IL-6 ↓ ,	H	[138]
			endotoxin tolerance ↓ , effector T cells ↑ , Treg↓	I	[54, 140, 141]
	SIRT2	AGK2	TNF-α ↓ , IL-1β ↓ , IL-6 ↓ , iNOS↓	H	[139]
Histone Methylation	EZH2	GSK343	TNF-α ↓ , IL-1β ↓ , IL-6 ↓ , organ injury↓	H	[145]
		3-DZNeP	survival ↑ , organ injury ↓ , MPO ↓ , TNF-α ↓ , IL-1β ↓ , IL-6 ↓ , circulating macrophages and neutrophils↓	H	[146]
Histone Demethylation	JMJD3	GSKJ4	TNF-α ↓ , IL-1β ↓ , IL-6 ↓ , IL-23a ↓ , IFN-β ↓ , CCL5 ↓	H	[43]
Histone Citrullination	PAD2	AFM32a	survival ↑ , NETs↓	H	[150]
	PAD4	Cl-Amidine	survival ↑ , organ injury ↓ , TNF-α ↓ , IL-1β ↓ , IL-6 ↓ , NETs↓	H	[147–149]
		GSK484	not improved		[150]

*H* hyperinflammatory stage, *I* immunosuppressive stage.

Nowadays, therapeutic interventions targeting histone methylation and regulatory enzymes are gradually being demystified. Enhancer of zeste homolog (EZH2), a catalytic component of histone methyltransferases, mediates H3K27me3 and has been found to participate in the regulation of immune dysfunction in sepsis [142, 143]. A prospective clinical study showed that EZH2 levels in lymphocytes could be used to assess the risk of mortality and secondary infection in patients [144], suggesting that targeting EZH2 be a potential therapeutic intervention. In a CLP mice model, using EZH2 inhibitor, GSK343, protects the intestine from septic injury [145]. In another study, researchers found that the use of 3-deazaneplanocin A, an EZH2 inhibitor, ameliorated mortality in the acute phase of septic mice by downregulating inflammatory cytokines and reducing the activation of circulating macrophages and neutrophils, which inhibited excessive inflammatory responses and organ damage in the acute phase of sepsis [146]. On the other hand, inhibiting the histone lysine demethylase JMJD3 also protects septic mice against early death caused by excessive inflammation [43]. As a small-molecule inhibitor of JMJD3, GSKJ4 relieves over-activated innate immunity [43].

Inhibitors targeting histone citrullination have recently received attention for their effects on the innate immune response to sepsis by inhibiting PAD and suppressing the formation of NETs. Use of the PAD4 inhibitor, Cl-Amidine, in septic murine models is shown to reduce the upregulated pro-inflammatory cytokines and enhance their overall survival [147, 148]. At the same time, Cl-Amidine acts as a protector against kidney injury induced by sepsis in rabbits [149]. Tian et al. reported that PAD2 selective inhibitor AFM32a also improves survival in septic mice through reduced inflammation and diminished NETs formation [150]. Whereas another small-molecule PAD4 inhibitor, GSK484, displays no survival improvement [150].

Using these enzymes’ inhibitors for histone modification in sepsis is still in the preclinical stage. Although some HDAC inhibitors have been approved for use in patients to treat hematologic malignancies, their impact on patients with sepsis is still unknown, including side effects, appropriate doses, *etc*. The clinical translation of these interventions in sepsis is potentially valuable. However, it is crucial to consider risks and toxicity and choose the appropriate timing for treatments based on the dynamic immune characteristics of the clinical patients.

## Conclusion and perspective

The role played by epigenetic regulation in the immunity of sepsis is complex. Histone modifications, as an essential component of epigenetic regulation, are important for regulating host immune status at different stages of sepsis, including the pro-inflammatory state after infection and the immunosuppressive state in the later stages of sepsis. With an in-depth exploration of this field, novel types of histone modification have been proposed, closely related to cellular metabolism. At the same time, this also implicates the mechanism of the link between metabolic reprogramming and immunity remodeling in sepsis. Different methods have been conducted to explore the function of histone modification in sepsis, including LPS stimulation, bacterial infection intraperitoneally, and CLP model. However, the evidence that elucidates different modeling methods directly impact histone modification in sepsis is limited. To the best of our knowledge, we compare different sepsis models and the differences of histone modification outcomes (Table 2). These have pioneered new horizons for discovering potential causes of immune remodeling in sepsis. Treatments for sepsis targeting various histone modification enzymes were developed and used in pre-clinical and clinical trials. However, the current knowledge of histone modifications is quite limited, so there are still many unsolved problems in this field that deserve further investigation. Targeting histone modifications to maintain immune status at a homeostatic level can be a valuable therapeutic option in the future. In conclusion, exploring the role played by histone modifications in immune remodeling during sepsis is critical for understanding the mechanism of sepsis and developing new treatments.

**Table 2:** Differences of histone modification between different methods induced sepsis.

Sepsis model	Histone site	Regulatory enzymes	Effects	ref
LPS stimulation	H3K4me3	SET1	Recruited SET1 upregulates H3K4me3 on promoter regions of p65 target genes and modulates inflammatory response.	[39]
		SYMD1	SYMD1 promotes H3K4me3 and increases IL-6 release in endothelial cells.	[40]
	H3K4me1		Upregulation of H3K4me1 promotes IFN-γ expression and mediates the generation of memory-like NK cells.	[113]
	H3K4/9me2		H3K4/9me2 demethylation regulates LSD1-BCL2L11/DUSP2 signaling and further limits IL-1β production.	[100]
	H3K27me3	JMJD3	JMJD3-induced H3K27me3 demethylation acts as a pro-inflammatory factor.	[42–44]
	H3k36me3		H3k36me3 promotes pro-inflammatory macrophage and enhances IL-1β production.	[99]
	H4K16ac	SIRT1	SIRT1 reduces H3K16 acetylation and inhibits TNF-α transcription during sepsis-induced inflammation.	[55]
	Histone3ac		The increased acetylated histones promoted the progression of sepsis by upregulating microRNA-29a to inhibit STAT3.	[57]
		HDACs	HDACs catalyzes the deacetylation of histone H3, which enables the activity of PAD4 and the formation of NETosis.	[109]
	H3K18Kla	p300	H3K18Kla promotes macrophage alternation from M1-like phenotype to M2-like.	[9]
Bacterial infection intraperitoneally	H3K27me3	JMJD3	H3K27me3 demethylation enhances pro-inflammatory cytokine IL-1β production as well as IL-6, TNF-α, and MCP-1 expression.	[43]
Cecal ligation and puncture (CLP)	H3K4/27me		Decreased H3K4me and upregulated H3K27me on GATA3 and TBX21 promotes CD4 + T cell inactivation and dysfunction.	[113]
	Histone ac	HDAC6	HDAC6-induced histone deacetylation might reduce B lymphocyte percentage.	[121]

*Methyltransferases* MLL1, SYMD1, *Demethylase* JMJD1, *Acetyltransferases* p300, *Deacetylases* HDACs, SIRT1.
